# Cognitive behavioral therapy for depression among adults in Japanese clinical settings: a single-group study

**DOI:** 10.1186/1756-0500-3-160

**Published:** 2010-06-07

**Authors:** Daisuke Fujisawa, Atsuo Nakagawa, Miyuki Tajima, Mitsuhiro Sado, Toshiaki Kikuchi, Motomi Hanaoka, Yutaka Ono

**Affiliations:** 1 Department of Neuropsychiatry, Keio University School of Medicine, 35 Shinanomachi, Shinjuku-ku, Tokyo, Japan; 2 Psycho-Oncology Division, National Cancer Center East, 6-5-1 Kashiwanoha, Kashiwa-shi, Chiba, Japan; 3 Stress Management Office, Keio University, 35 Shinanomachi, Shinjuku-ku, Tokyo Japan; 4 Department of Neuropsychiatry, Tokyo Women's University School of Medicine, 8-1Kawatacho, Shinjuku-ku, Tokyo, Japan; 5 Health Management Center, Keio University, 35 Shinanomachi, Shinjuku-ku, Tokyo Japan

## Abstract

**Background:**

Empirical support for cognitive behavioral therapy (CBT) for treating Japanese patients with major depression is lacking, therefore, a feasibility study of CBT for depression in Japanese clinical settings is urgently required.

**Findings:**

A culturally adapted, 16-week manualized individual CBT program for Japanese patients with major depressive disorder was developed. A total of 27 patients with major depression were enrolled in a single-group study with the purpose of testing the feasibility of the program. Twenty six patients (96%) completed the study. The mean total score on the Beck Depression Inventory-II (BDI-II) for all patients (Intention-to-treat sample) improved from 32.6 to 11.7, with a mean change of 20.8 (95% confidence interval: 17.0 to 24.8). Within-group effect size at the endpoint assessment was 2.64 (Cohen's d). Twenty-one patients (77.7%) showed treatment response and 17 patients (63.0%) achieved remission at the end of the program. Significant improvement was observed in measurement of subjective and objective depression severity (assessed by BDI-II, Quick Inventory of Depressive Symptomatology-Self Rated, and Hamilton Depression Rating Scale), dysfunctional attitude (assessed by Dysfunctional Attitude Scale), global functioning (assessed by Global Assessment of Functioning of DSM-IV) and subjective well-being (assessed by WHO Subjective Well-being Inventory) (all p values < 0.001).

**Conclusions:**

Our manualized treatment comprised of a 16-week individual CBT program for major depression appears feasible and may achieve favorable treatment outcomes among Japanese patients with major depression. Further research involving a larger sample in a randomized, controlled trial design is warranted.

**Trial registration:**

UMIN-CTR UMIN000002542.

## Background

The 12-month prevalence of mood disorder in Japan is 3.1% and is associated with higher incidence of suicide and marked economic morbidity [1]. Therefore, it is important for clinicians as well as policy makers to establish quality treatment programs for major depression.

Thanks to the robust research evidence on its efficacy for treating depression [2-4], cognitive behavioral therapy (CBT) has been drawing considerable attention among the general public [5], as well as among clinicians, in Japan. The number of members of the Japanese Association for Cognitive Therapy (JACT) has been quadrupled from approximately 300 in 2001 to 1400 in 2009 [6].

Despite these developments, CBT has yet to be adopted in daily clinical practice in Japan. A recent nationwide survey demonstrated that only 28% of all the medical facilities in Japan were satisfied with their delivery of psychotherapy, and CBT was listed as the 'most in need' psychotherapy among the various types of psychotherapy [7]. Insufficient provision of CBT in daily practice is largely attributable to lack of empirical support for CBT among Japanese depression patients. The models and therapeutic components of CBT were mostly developed based on Western conceptualizations of depression, and most studies demonstrating the efficacy of CBT were conducted in Europe and North America [2,3]. Empirical support for CBT in Japanese clinical settings is still required.

Literature review and our clinical experience suggest that Japanese patients generally prefer directive, problem-solving approaches, where the therapists are required to make the therapy more structured and solution-focused [8,9]. Compared with Western cultures, more emphasis is placed on interpersonal relationships than self-fulfillment or self-development [10]. There are needs for consideration of family as an essential part of the treatment. The Japanese culture has formerly been family- and community- oriented, but recently there is a shifting trend for increasing emphasis on individuality. These issues should be comprehensively addressed during the therapy.

On the basis of this information, we developed an individual CBT program for treating Japanese patients with depression. The program was constructed upon the model developed by Beck et al. [11], with some adaptation to address the cultural characteristics of the Japanese patients. The treatment manual is downloadable from the website of the Japanese Ministry of Health, Labor and Welfare [12]. The overview of the program is shown in the Table 1. Problem-solving techniques and interpersonal issues were emphasized and the therapists were encouraged to refer to the relevant chapters whenever considered necessary. Furthermore, the therapists were encouraged to give feedbacks to the patients about the case conceptualization in the earlier phase of the therapy.

**Table 1:** Overview of stages in therapy.

Stage	Session	Purpose	Agenda	Tools/homework
1	1 - 2	Building alliance Psychoeducation Motivate the patient Socializing the patient	Review on symptoms, course of illness and developmental history Psychoeducation on depression, cognitive models, structure of the therapy	"What is depression?" "What is CBT?"

2	3 - 4	Case conceptualization Goal setting Activating the patient	Collaboratively setting treatment goals Activity scheduling Brief feedback on case conceptualization	Problem list Activity record

3	5 - 6	Identifying mood and automatic thoughts	Dysfunctional thought record (triple column)	"How to identify your moods and thoughts"

4	7 - 12	Testing automatic thoughts (Optional - dissolving interpersonal conflicts/problem solving)	Dysfunctional thought record (seven columns) (Optional module - assertive training/problem solving)	"How to balance your thoughts" Interpersonal module Problem-solving module

5	13 - 14	Identifying schemas	Dysfunctional thought record Discussion on schemas	"Rules of your mind"

6	15 - 16	Termination Relapse prevention	Review of the therapy Relapse prevention Preparation for booster sessions	"Upon ending your therapy"

To the best of our knowledge, no study to date in Japan has examined the feasibility of a manualized individual CBT for depression. Therefore, the present pragmatic study aimed to test feasibility of our manualized CBT program for major depressive disorder in outpatient settings in Japan. We examined its feasibility by exploring the acceptability of intervention with treatment providers and patients, rate of complete/dropout of treatment, resources required to carry out the intervention and effect of treatment.

## Methods

### Participants

Participants were the ambulatory patients aged 18 - 60 years, who met the criteria for a primary diagnosis of current Major Depressive Disorder (MDD) according to the Structured Clinical Interview for Axis-I DSM-IV Disorders [13]. The exclusion criteria were active suicidal intent, unstable physical conditions, or antisocial personality disorder. The participants were recruited through referral by psychiatrists at the participating study sites between April 2004 and August 2007. The study sites consisted of two psychiatric hospitals, two university hospitals and one psychiatric clinic in Tokyo. These sites were selected because they had expertise in both treating depression and providing psychotherapy.

### Interventions

The individual CBT program described above was provided on a weekly or fortnightly basis. The therapy comprised of 16 sessions, with each session lasting for 50 minutes. The therapists were allowed either to extend the therapy to a maximum of 20 sessions, or to abort the program after the seventh session upon agreement between the patient and the therapist, provided that the Beck Depression Inventory (BDI-II) had fallen to the score of 13 or below (remission). The therapists were psychiatrists or master's-degree clinical psychologists with over 3-year clinical experience and with experience in CBT of 1-5 years. The therapists participated in a two-day workshop to ensure treatment fidelity, and afterward participated two-hour fortnightly group supervision sessions. The group supervision was lead by one of our co-authors (YO), the founder of the JACT and a fellow of Academy of Cognitive Therapy [14], facilitating discussion of therapeutic difficulties and impasses, providing skills acquisition, and peer support and interaction. The supervisee-therapists were asked to make presentation regarding changes in the BDI-II scores, treatment process, case formulation, and treatment planning. A therapist's self-report check list outlining the key elements of the treatment, which was originally developed for this study, was used to ensure treatment adherence.

The patients were allowed to continue the antidepressants and anxiolytics that had been prescribed at the baseline throughout the study.

### Outcome measures

The primary outcome measure was the patients' subjective severity of depression which was assessed using the Beck Depression Inventory (BDI-II) [15]. The severity of depression is categorized based on the following BDI-II scores in Japanese samples: a score of 13 or less as minimal (or remission); 14 to19 as mild; 20 to 28 as moderate; 29 or greater as severe [16].

The secondary outcomes included the 17-item Hamilton Rating Scale for Depression (HAMD-17) [17], the Global Assessment of Functioning (GAF) scale of DSM-IV [18], the World Health Organization Subjective Well-being Inventory (SUBI)[19], and the 24-item Dysfunctional Attitude Scale (DAS-24) [20], with the purposes of assessing objective depression severity, global functioning, subjective well-being, and dysfunctional attitudes, respectively. The 16-item Quick Inventory of Depressive Symptomatology Self-Rated (QIDS-SR) [21] was also administered in order to facilitate the comparison of the results with a variety of past studies.

The BDI-II was administered at each session, and other measurements were administered at pre- and post- treatment. The HAMD-17 was administered by the study therapists. A training workshop was conducted in order to ensure the inter-rater reliability of the measures. The raters were certified when 80% accuracy was achieved, as referring the demonstration videos. The measures were not blinded to the treating clinician, because monitoring of social and psychological status was considered to be an essential part of the CBT program.

### Statistical analysis

The analysis was by intention-to-treat (ITT), and for non-completers, the last obtained data were carried forward into the endpoint assessment. The baseline and endpoint scores on the outcome measures were compared using t tests. Regarding the BDI-II, changes from the baseline at each visit was analyzed using repeated ANOVA. The cutoff point for remission was defined as an endpoint BDI-II score of 13 or less [16], and treatment response was defined as a reduction of 50% on the BDI-II [22]. Regarding the HAMD-17, remission was defined as an endpoint total score of 7 or less, and treatment response was defined as a reduction of at least 50% [23].

All statistical tests were two-tailed, and an alpha value of less than 0.05 was considered statistically significant. Within-group effect sizes were computed as d, the standard mean difference. Cohen [24] defines effect sizes as small (d = 0.20), medium (d = 0.50), and large (d = 0.80). All data analyses were conducted using SPSS version 16.0J software (SPSS Inc., Chicago, IL, USA).

### Ethical considerations

All participants gave written informed consent after receiving a full description of the study. The protocol of this study was approved by the Ethics Committee at each study site, and was registered in the national UMIN Clinical Trials Registry (ID: UMIN000002542).

## Results

### Treatment acceptability by the therapists

All the participating therapists completed the training program and were able to adhere to the treatment protocol under the supervision.

### Baseline data

A total of 27 who met the inclusion criteria patients were referred to the study. All of them submitted a written informed consent and entered the study. Of those, 19 patients were diagnosed with recurrent MDD and the remainders were diagnosed with single episode MDD. Three patients had one of the following concurrent psychiatric disorders: panic disorder, borderline personality disorder and schizotypal personality disorder. Other clinical characteristics of the participants are shown in Table 2.

**Table 2:** Clinical characteristics of participants.

Characteristics	n	%
Socioeconomic characteristics		
Gender		
Female	18	66.7
Male	9	33.3
Marital status		
Single	13	48.2
Married or cohabitant	10	37.0
Divorced	4	14.8
Education		
Finished tertiary education	12	44.4
Employment		
Employed	20	74.1
Housewife	3	11.1
Unemployed	4	14.8
		
Clinical characteristics		
Major Depressive Disorder, single episode	8	29.7
Major Depressive Disorder, recurrent episode	19	70.3
		
Concomitant antidepressants or anxiolytics use		
Yes	22	81.5
No	5	18.5
	
	Mean	SD
	
Age (years)	38.5	9.5
Duration of current depression episode (years)	5.6	4.3
Range (years)	1 to 13	

### Treatment and dropout

**Figure 1:**
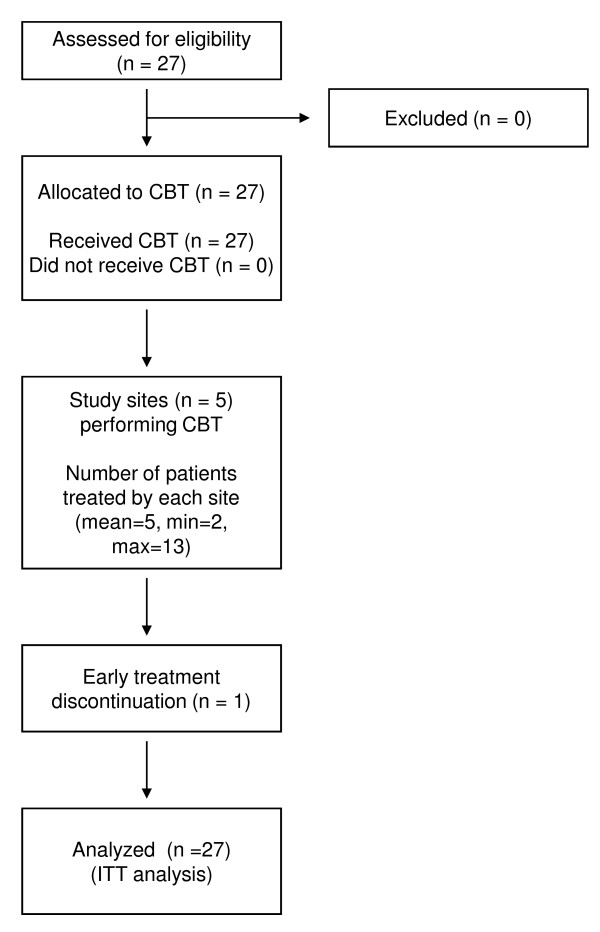
**Flow of the participants**. CBT: cognitive behavioral therapy. ITT: intent-to-treat. Min: minimum. Max: maximum.

Twenty-six patients (96%) completed the program. One female patient with borderline personality disorder dropped out at the fifth session. The mean number of attended CBT sessions was 15.3 (SD = 2.8). The flow of participants through each stage is shown in Figure 1.

### Primary Outcome of the Program

**Figure 2:**
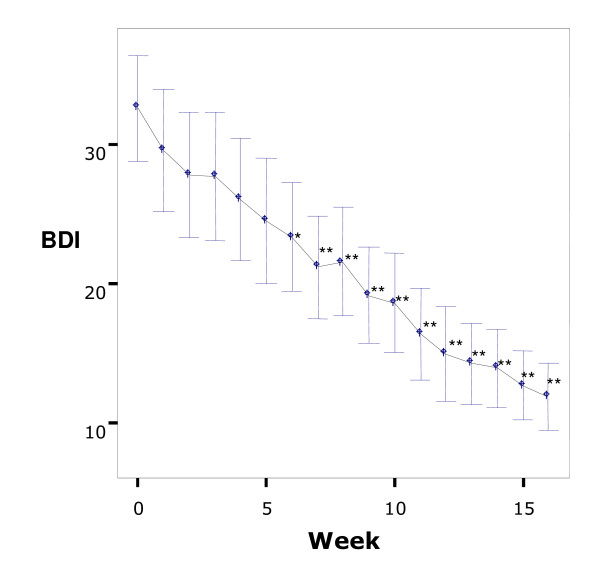
**Changes over time in mean Beck Depression Inventory-II scores**. Intent-to-treat sample. Comparison with baseline score, *p < 0.05, **p < 0.01. Bars: 95%Confidence Interval.

A significant improvement on the BDI-II scores was observed from the baseline to week 16 (p < 0.001) (Table 3). The mean total score of BDI-II decreased from 32.6 to 11.7, which corresponds to clinical improvement from severe to minimal depression. Twenty one patients (77.7%) were judged to be treatment responders and 17 patients (63.0%) were judged to be remitters. The mean score on the BDI-II continuously decreased as sessions proceeded and it became significantly lower after the fifth session (p < 0.05) (Figure 2).

**Table 3:** Comparison of pre- and post-treatment scores of outcome measures.

	Baseline		Endpoint		% improvement	Cohen's *d*
			
Outcome Measures	Mean	SD	Mean	SD		
BDI-II	32.6	9.5	11.7*	5.9	64.1	2.64
HAMD-17	24.3	7.4	8.1*	3.4	66.6	2.81
QIDS-SR	21.3	7.1	9.4*	3.4	19.9	2.14
GAF	46.1	9.6	73.2*	8.5	57.8	2.99
DAS-24	106.4	29.6	85.5*	14.7	19.9	0.89
SUBI - health subscale	38.4	6.4	38.3*	5.0	20.8	0.02
SUBI - fatigue subscale	32.6	9.5	46.4*	5.4	64.1	1.79

Intent-to-treat sample, Last-observation-carried forward analysis

BDI-II: Beck Depression Inventory-II score

HAMD: Hamilton Depression Rating Scale

QIDS-SR: Quick Inventory of Depressive Symptomatology -Self Rated

GAF: Global Assessment of Functioning of DSM-IV

DAS: Dysfunctional Attitude Scale

SUBI: WHO Subjective Well-being Inventory

* p < 0.001

### Secondary Outcomes of the Program

Table 3 shows the means and standard deviations of all other measures at pre- and post-treatment. Depression severity measures (HAMD-17 and QIDS-SR), dysfunctional attitudes (DAS-24), subjective well-being (SUBI) and social functioning (GAF) showed significant improvement after the program (all p values < 0.001). On the HAMD-17, 12 patients (44%) were judged to be treatment responders and 9 patients (33%) were judged to be remitters.

## Discussion

The result of this 16-week individual CBT program offers preliminary optimism for applying CBT for treating major depressive disorder in Japan. Favorable outcomes were demonstrated in regard to excellent acceptability by both of the treatment providers and the patients, low dropout rate, and improvement in pervasive outcome measures. The additional resources to provide the treatment are minimal, except that the therapists need formal training for CBT under regular supervision.

The proportions of the patients who remitted and/or responded are comparable with those of the Sequenced Treatment Alternatives to Relieve Depression study (STAR*D) [25], one of the largest pragmatic clinical trials for major depression involving CBT, which established a benchmark for acceptable dropout rate and effectiveness. The dropout rate in the STAR*D was 9.2% for its CBT augmentation upon medication arm, whereas the dropout rate in the present study was 3.7%. The STAR*D reported a response rate of 35.4% and a remission rate of 30.8% on the self-rating measure, whereas the present study showed a response rate of 77.7% and a remission rate of 63.0%. The effect size of 2.64 observed for the BDI-II is comparable with the results of past CBT studies, in which effect sizes of 1.82 - 2.43 were reported [26,27].

However, we must be cautious about the interpretation of our data, because the present study contains the following limitations. First, this was a single-arm study without a control group; therefore we cannot be conclusive that our CBT program was effective. The Hawthorne effect, which is typical seen in uncontrolled studies, cannot be denied in our study design, and therefore, future randomized controlled study is required. Although the effect size was high, it might have been inflated due to the lack of control group [28]. Second, the study involved a limited number of patients. The distribution of the participating patients was somewhat skewed from the distribution of the national statistics. Relatively smaller proportion of patients who were widowed or divorced, and who were unemployed participated in this study. Third, CBT was delivered by the physicians in charge of their care for most of the participants; therefore, the positive therapeutic bonds formed prior to the study may have contributed to the low dropout rate and high improvement rate. Fourth, the treatment adherence was monitored based on the therapists' presentation and therapy record, not by audio-taped record. Finally, the HAMD-17 was evaluated by the therapists and its reliability is compromised.

These limitations may be off-set by the following factors. First, we employed a self-report measure (BDI-II) as the primary outcome. Although the participants may have rate their severity of depression lower due to bias towards the treating clinician, submitting a self-report measure of depression severity is an essential part of CBT program. Second, statistically significant improvement was demonstrated on multiple measures, with the patients' self-ratings showing a similar magnitude of improvement as compared with the clinician-rated measures. Third, baseline severity of depression ranged from mild to severe depression, which is considered to be representative of the general Japanese clinical population. Fourth, the study site comprised of facilities of different characteristics (including university hospital, mental hospital and mental clinic).

The favorable outcome for CBT observed in this study is an important first step for the implementation of CBT in Japanese clinical settings. It may also have a potential impact to therapists outside Japan who treat depression patients of Asian ethnicity. To date, clinical trials of CBT for depression among Asian population have been only sporadically reported; therefore, our report provides useful informative findings in this issue. Our findings support the applicability of CBT for various cultures, consistent with findings in non-Western studies from Hong Kong [29] and Korea [30].

## Conclusions

It is indicated that this cognitive behavioral intervention can lead to positive outcomes in treating Japanese patients with major depressive disorder, although further controlled trials which address the limitations of this study are required.

## Competing interests

The authors declare that they have no competing interests.

## Authors' contributions

DF designed and managed the study and drafted the manuscript. AN assisted with drafting the manuscript. AN and MT performed the statistical analyses. MS, TK and MH coordinated the study. YO participated in the study coordination and provided supervision to the therapists. All the authors read and approved the final manuscript.
